# Supplementary material for: A Critical View of the Application of the APEX Software (Aqueous Photochemistry of Environmentally-Occurring Xenobiotics) to Predict Photoreaction Kinetics in Surface Freshwaters
Source: Molecules. 2019 Dec 18;25(1):9. doi: 10.3390/molecules25010009 (PMC7017383; doi:10.3390/molecules25010009)
Supplement: Supplementary file 1 [file molecules-25-00009-s001.zip › 3.2.4_gcc-4.4.0.zip!/3.2.4_gcc-4.4.0/3.2.4_gcc-4.4.0/doc/octave/html/interpreter/String-Input-Conversions.html]

String Input Conversions - Untitled


Next: Binary I/O,
Previous: Numeric Input Conversions,
Up: C-Style I/O Functions


---

#### 14.2.15 String Input Conversions

This section describes the `scanf` input conversions for reading
string and character values: %s and %c.

The %c conversion is the simplest: it matches a fixed number of
characters, always. The maximum field with says how many characters to
read; if you don't specify the maximum, the default is 1. This
conversion does not skip over initial whitespace characters. It reads
precisely the next n characters, and fails if it cannot get that
many.

The %s conversion matches a string of non-whitespace characters.
It skips and discards initial whitespace, but stops when it encounters
more whitespace after having read something.

For example, reading the input:

```
      hello, world
```

with the conversion %10c produces `" hello, wo"`, but
reading the same input with the conversion %10s produces
`"hello,"`.
